# Prevalence of Imaging-Detected Silent Female Breast Cancer in Autopsy Specimens: A Study Using Image-Guided Biopsies

**DOI:** 10.7759/cureus.32776

**Published:** 2022-12-21

**Authors:** Zacharoula Sidiropoulou, Ana Vasconcelos, Cristiana Couceiro, Carlos Santos, Ana Virginia Araujo, Inês Alegre, Cláudia Santos, Filipa Campos Costa, Diogo Cardoso, Vasco Cardoso, Rita Sampaio, Fátima Cardoso, Pere Gascón

**Affiliations:** 1 Breast Unit, Centro Hospitalar Lisboa Ocidental, Lisbon, PRT; 2 Doctoral School of Biomedicine, Faculty of Medicine and Health Sciences, University of Barcelona, Barcelona, ESP; 3 Radiology, Centro Hospitalar Lisboa Ocidental, Lisbon, PRT; 4 Medicina Legal, Instituto Medicina Legal e Ciências Forenses, Delegação Sul, Lisbon, PRT; 5 General Surgery, Centro Hospitalar Lisboa Ocidental, Lisbon, PRT; 6 General Surgery, Centro Hospitalar Lisboa Ocidental, Lisboa, PRT; 7 Pathology, Centro Hospitalar Lisboa Ocidental, Lisbon, PRT; 8 Breast Unit, Champalimaud Foundation, Lisbon, PRT; 9 Laboratory of Molecular and Translational Oncology, CELLEX, Barcelona, ESP

**Keywords:** forensic autopsy, breast cancer screening, mammography, overdiagnosis, silent breast cancer

## Abstract

Background

This study was conducted to evaluate the prevalence of imaging-detected silent breast cancer in females, with the hypothesis that the incidence of imaging-detected silent breast cancer in females is greater than the true disease incidence. The main purpose of this study is the attempt to prove whether breast imaging can identify silent breast cancers that apparently are common in serial histology analysis.

Methodology

A series of 217 consecutive medicolegal autopsies on fresh Portuguese cadavers were performed from July 2016 to December 2019 at the National Institute of Legal Medicine and Forensic Science, Lisbon, Portugal. The criteria for exclusion were age younger than 40 years, the autopsy performed in less than 48 hours after death, any major injury to one or both breasts, and known or clinically evident breast cancer. Once the eligibility criteria were met, and the sample collection authorization was obtained, a bilateral subcutaneous modi­fied radical mastectomy was performed in each fresh cadaver at the National Institute of Legal Medicine and Forensic Science. Mammography, ecography, and excisional biopsies of suspect areas were conducted on the collected samples.

Results

The indication for excisional biopsy by imaging was assigned in eight cases, and no breast cancer was discovered in the excised specimens.

Conclusions

In light of the findings, it cannot be concluded that the imaging-detected silent breast cancer prevalence is higher than the actual incidence of the disease, so the author’s initial hypothesis was rejected. Mammography does not overdiagnose breast cancer. Benign breast alterations are common, accounting for 43.6% of the corpses collected, while low-suspicion alterations were discovered in 1.84% of breast samples. The objective examination, which included inspection and palpation, missed 37.5% of the biopsied breast changes. This finding indicated that an objective examination leads to a significant number of false-negative results which cannot be used as a screening method.

## Introduction

Globally, breast cancer is the most common malignancy among women, with an increased incidence and high mortality [1]. Worldwide, breast cancer prevalence varies considerably as a result of differences in age distribution, diet, lifestyle, ethnicity, genetic background, and other breast cancer risk factors among the different populations. The same variation can be found in the European context, with incidence rates varying from 49 to 148 new cases per 100,000 women [2]. On the other hand, the differences in mortality rate present a minor variability from 15 to 36 new cases per 100,000 women. In Portugal, the incidence and mortality trends mirror the European figures, with incidence and mortality rates of 118.5 and 30.4 cases per 100,000 women, respectively, as per the statistics provided by the 2019 report of the Directorate-General of Health (https://www.sns.gov.pt/wp-content/uploads/2020/09/Relatorio_Anual_Acesso_2019.pdf). The national screening program covers 67.70% of the target population, with an adhesion rate of 60.89%, which is considered insufficient for an adequate screening program. Breast cancer incidence and mortality patterns vary significantly even within the country and among different regions. Further, the capital of Portugal (Lisbon) only lately started officially screening, with most of the population followed in private or general practice settings. Although it is believed that the screening programs (opportunistic or organized programs) enable the detection of breast cancer in the earlier stages, the incidence of advanced metastatic breast cancer remains stable (10‑15% of breast cancers in Portugal are diagnosed at stage IV) [3]. Therefore, it is believed that screening and novel therapies are equally important in reducing mortality [3]. Given the controversy regarding the ideal modality of breast screening, in terms of means and periods, this study was designed to investigate if mammography constitutes an imaging technique that can over-detect malignant lesions, thus overdiagnosing breast cancer. The aim was to quantify the cases of existing cancers that had not clinically manifested. A thorough literature research revealed only five medicolegal (and not hospital) autopsy studies [4-8] designed to define the natural reservoir of the disease. All of these studies focused on the serial histological analysis of the breast tissue, with a resulting high volume of premalignant and in situ lesions, although no real correlation was noted over the fact that it was not mammography that detected them. Although it has been concluded by Nielsen et al. [6] that “to definitively characterise the ductal carcinoma in situ (DCis) reservoir, a large prospective study of the age‑specific prevalence of occult breast cancer is sorely needed,” hardly any studies have been performed since 1987 despite controversies surrounding breast cancer screening and eventual overdiagnosis.

## Materials and methods

The samples comprising the study population were obtained from the National Institute of Legal Medicine and Forensic Science in Lisbon, following a proper tissue collection. The study employed Cochran’s [9] sample size estimation procedure where the target population was infinite. Therefore, the sample size was calculated at a 95% confidence interval, 0.12 proportion, and to achieve the null hypothesis with a precision level of 0.05, an estimated population size of 182 cadavers was required. The study group consisted of a series of consecutive medicolegal autopsies on fresh Portuguese cadavers performed from July 2016 to December 2019 at the National Institute of Legal Medicine and Forensic Science, Lisbon, Portugal.

The criteria for exclusion were age younger than 40 years, the autopsy performed in less than 48 hours after death, major injury to one or both breasts, and known or clinically evident breast cancer. Once the eligibility criteria were met, and the sample collection authorization was obtained, a bilateral subcutaneous modified radical mastectomy (bsMRM) was performed through a Douformentel incision (allowing the subsequent reconstruction, previous to the release of the corpse) in each fresh cadaver at the National Institute of Legal Medicine and Forensic Science. The characteristics of the cadavers, such as age, height, weight, and body mass index (BMI), were collected from the referring file, while medical history data was not included due to inadequate pre-study collection.

Each specimen was properly identified by spatial orientation and, after conditioning in sealed bags, was transported within an appropriate container to the Hospital São Francisco Xavier (Lisbon, Portugal) and submitted to measurement (three-dimensional), waiting, inspection, palpation, ultrasound, and mammography by breast radiologists and breast surgeons. The collected tissues were imaged using the GE Healthcare digital mammography system, Senographe Essential™ (GE Healthcare BioSciences, Pittsburgh, PA, USA), with an X-ray beam of 27 kV (range: 60-70 mA) and 10 15 decanewtons (daN) compression, depending on tissue density and size. The visualization screen had a resolution of 5 megapixels (GE Healthcare LOGIQ™ S7 Expert ultrasound system, with a medium frequency of 9 15 MHz; GE Healthcare BioSciences, Pittsburgh, PA, USA).

Breast tissue, classified with the Breast Imaging Reporting and Data System (BI-RADS) category three or higher, was submitted to wire-guided or direct excisional surgical biopsy. According to the fifth edition of the ACR BI-RADS Atlas, ACR BI-RADS [10] system was used. In the pre-analytical phase, breast biopsies were fixed in 10% buffered formalin (JTBaker) for 24 hours, and lumpectomy specimens were fixed for 48-72 hours at room temperature (20°C). Formalin-fixed, paraffin-embedded (VWR International, EUA) tissues were processed in Sakura’s Tissue-Tek VIP and cut into 3 µm sections, one cut per adhesive slide (Superfrost Plus Gold, Thermo Scientific, EUA), with respective positive control. Tissue section adhesion time and temperature were held constant for one hour at 70°C. Following these procedures, the slide was subjected to labeling using the immunocytochemistry (ICC) method. The ICC panel of primary antibodies used against Ki67 (clone 30-9, Cat. 790-4286), estrogen receptor (ER) (clone SP1, Cat. 790-4324), and progesterone receptor (PR) (clone 1E2, Cat.790-2223) were performed in the BenchMark ULTRA using Optiview DAB IHC Detection Kit (Cat. 760-700), for Ki67 and Ultraview Universal DAB Detection Kit (Cat. 760-500) for ER and PR (Ventana Medical Systems, Tucson, AZ, USA). The slides were observed by a surgical pathologist under an optical microscope.

This study was approved by the Ethics Committee for Health (CES) of Centro Hospitalar de Lisboa Ocidental (approval number: 20170700050). The study protocol was registered in the ClinicalTrials.gov database (registry ID: NCT02480933).

## Results

**Figure 1:**
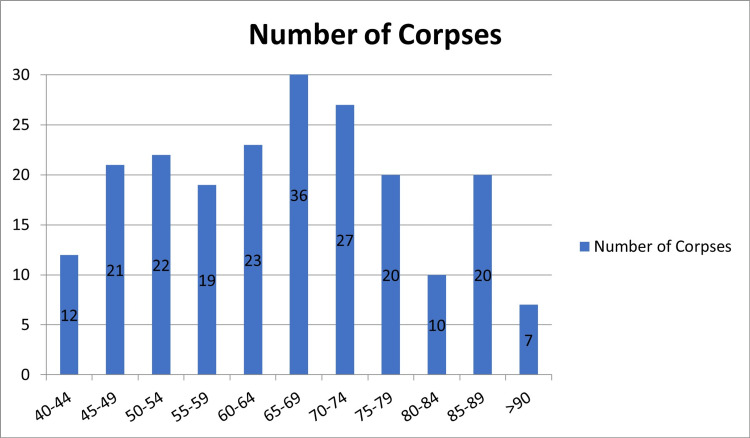
Age distribution of the corpses.

A total of 217 cases were submitted to bsMRM and proceeded to tissue evaluation. The average postmortem to biopsy duration was 18 hours. Age at the time of death ranged from 40 to 91 years, with a mean age of 65.53 years (Figure 1).

The mean BMI was 24.89 kg/m^2^. Out of the 271 cadavers, 94% were of Caucasian, and 6% were of Black ethnicity. In total, 65 (31.08%) of the 271 patients died unexpectedly from myocardial infarction acute heart failure (Table 1).

**Table 1:** Findings at the time of death.

Findings at death	Number of corpses
Acute myocardial infarction	65
Acute cerebrovascular accident	45
Hypovolemic shock	20
Viral pneumonia	16
Head trauma subarachnoid hemorrhage	14
Hypoxic encephalopathy	8
Poisoning	8
Aspiration	7
Acute alcohol intoxication	4
Acute pulmonary embolism	4
Asphyxiation	4
Right colon adenocarcinoma	3
Diabetic ketoacidosis	2
Lung adenocarcinoma	2
Peritonitis	2
Peritonitis post-left hemicolectomy	2
Viral meningitis	2
Bacterial meningitis	1
Bacterial pneumonia	1
Gastric adenocarcinoma perforation	1
Hepatic metastasis of left colon adenocarcinoma	1
Hypertrophic cardiomyopathy	1
Left colon adenocarcinoma perforation	1
Left colon metastatic adenocarcinoma	1
Ovarian metastatic adenocarcinoma	1
Peritonitis post-right hemicolectomy	1

**Figure 2:**
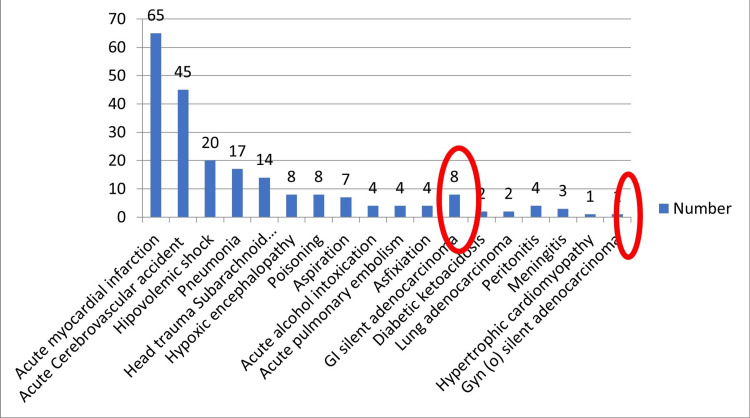
Findings at the time of death.

Interestingly, seven gastrointestinal tract silent (diagnosed at the autopsy) adenocarcinomas (six colon and once gastric carcinomas) and one silent ovarian adenocarcinoma were diagnosed (Figure 2).

**Figure 3:**
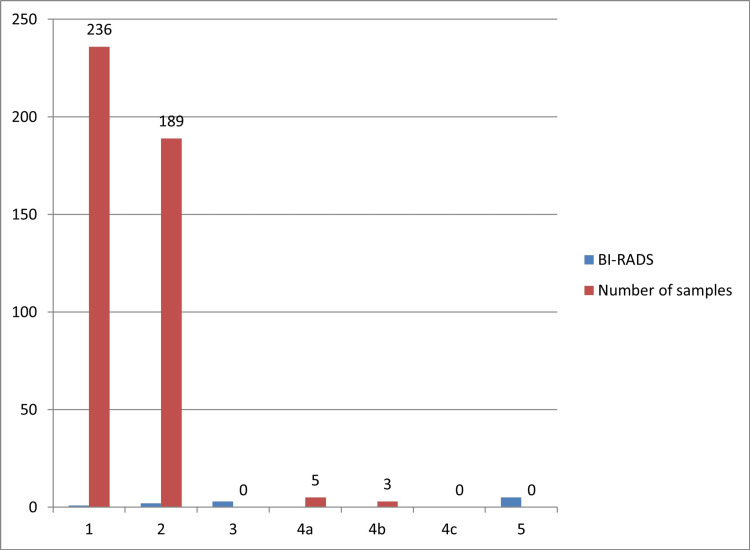
Samples and their BI-RADS classification. BI-RADS = Breast Imaging Reporting and Data System

No breast cancer was detected. There was no history or scars from breast surgery, nor did the samples have a confirmed diagnosis or clinical signs of breast cancer. The mean breast tissue weight processed was 2,005.244 g/cadaver, and the dimensions were: mediolateral 25.97 cm, superoinferior 22.87 cm, and anteroposterior 3.39 cm per tissue. Moreover, it appeared that there was a weak correlation between BMI and breast tissue weight (correlation index of 0.076 and covariance index of 277.836). In volumetric terms, the breast tissue was submitted for imaging, and to approximate its shape to a hemi-ellipsoid, the following calculus was applied: ellipsoid dimensions and ellipsoid formula (Knud Thomsen) divided by two. The total breast tissue volume was 836,821.9 cm^3^ (836,822 L). The total volume of breast tissue created was 836,821.9 cm^3^ (836,822 L). Breast volume and BMI did not appear to correlate (correlation index of 0.008). Breast density and BMI appeared to have a minor correlation, with a correlation index of 0.02 and a covariance index of 0.09. BI-RADS classification found changes in one in 236 (54.50%), two in 189 (43.6%), and three in 0 (0%). If BI-RADS 3 results were detected, they were categorized as 4a and subjected to biopsy because there is no probability of a six-month control; 4a in five (1.15%), and 4b (0.69%) in three breast samples (Figure 3).

In general, as shown in Table 2, an objective examination (OE), that is, inspection and palpation, even if performed by a dedicated breast surgeon, cannot be used to detect breast alterations.

**Table 2:** BI-RADS and objective examination. BI-RADS = Breast Imaging Reporting and Data System; OE = objective examination

BI-RADS	Normal OE	Pathologic OE
1	236	0
2	209	1
4a	2	3
4b	1	2

There was one radiologic false-positive result, pathologic OE with no imaging correspondence (0.23% more biopsies), and three radiologic false-negative results, normal OE where biopsy had been performed for imaging alterations of 4a and 4b (missing the remaining 37.5% of breast changes). This finding points out that an OE with a high false-negative rate cannot be used as a screening method.

The mammographic analysis of the samples revealed radiologic benign microcalcifications in 42 cases, 35 dispersed and seven localized. Moreover, benign macrocalcifications were detected in 13 cases, mostly localized in the upper quadrants. Further, in 28 cases, both types of radiologic benign calcifications were present. Additionally, plasma cell mastitis was found in eight cases.

Ecography revealed cysts in 14 cases, ductal ectasia in 13 cases, both types of lesions in eight cases, lipomas in three cases, and steatonecrotic lesions in five cases. Moreover, six cases had benign axillary adenomegalies, and two had intramammary adenomegalies. The excisional biopsies concerned 4a (five cases) and 4b BI-RADS (three cases) classification changes.

Biopsied cadavers

**Figure 4:**
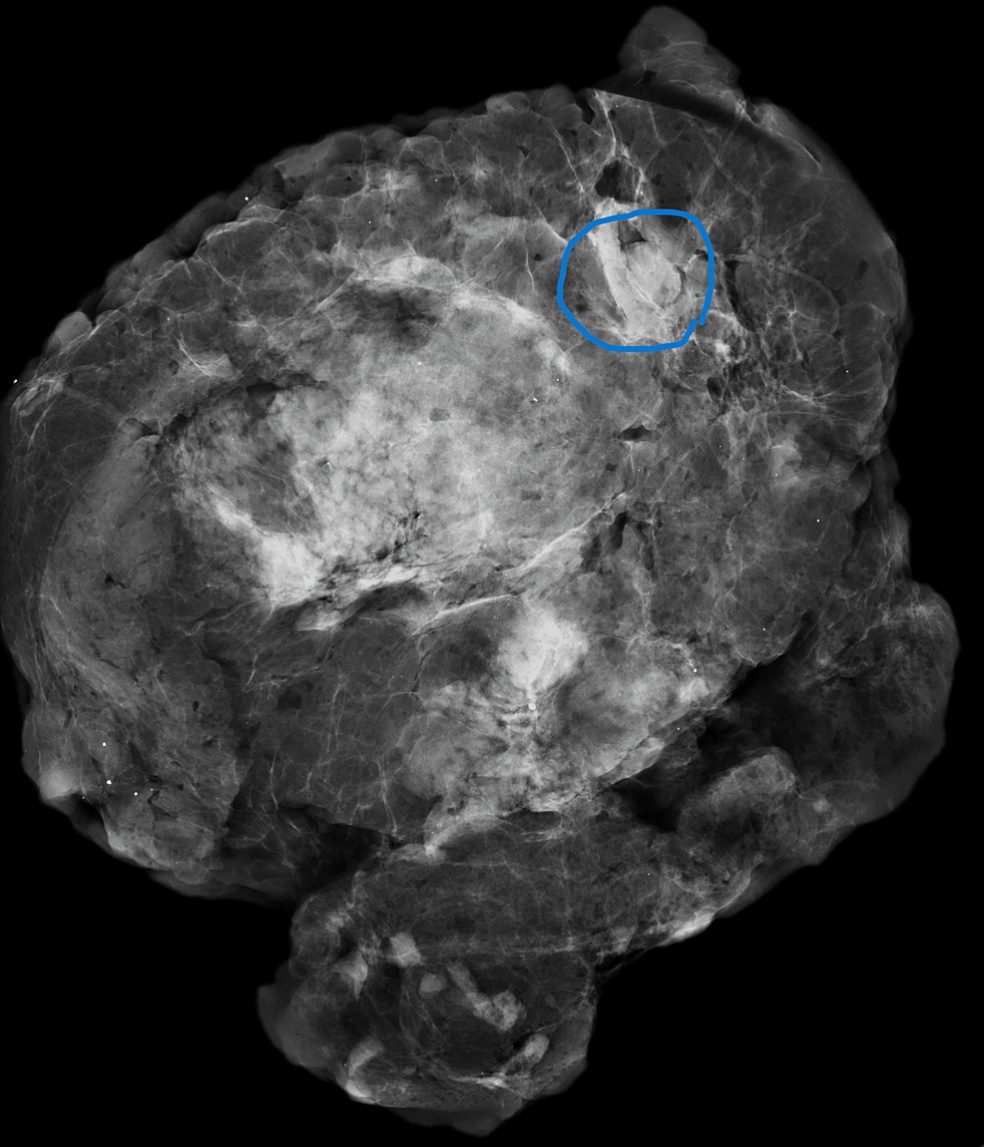
Case one mammogram showing a 25 mm ill-defined lesion in the upper quadrants.

Case one: A 42-year-old Black female with a pathologic left breast palpation and 4b BI-RADS due to a 25 mm ill-defined lesion in the upper quadrants. Histologically, there were fibrocystic changes in the area (Figure 4).

**Figure 5:**
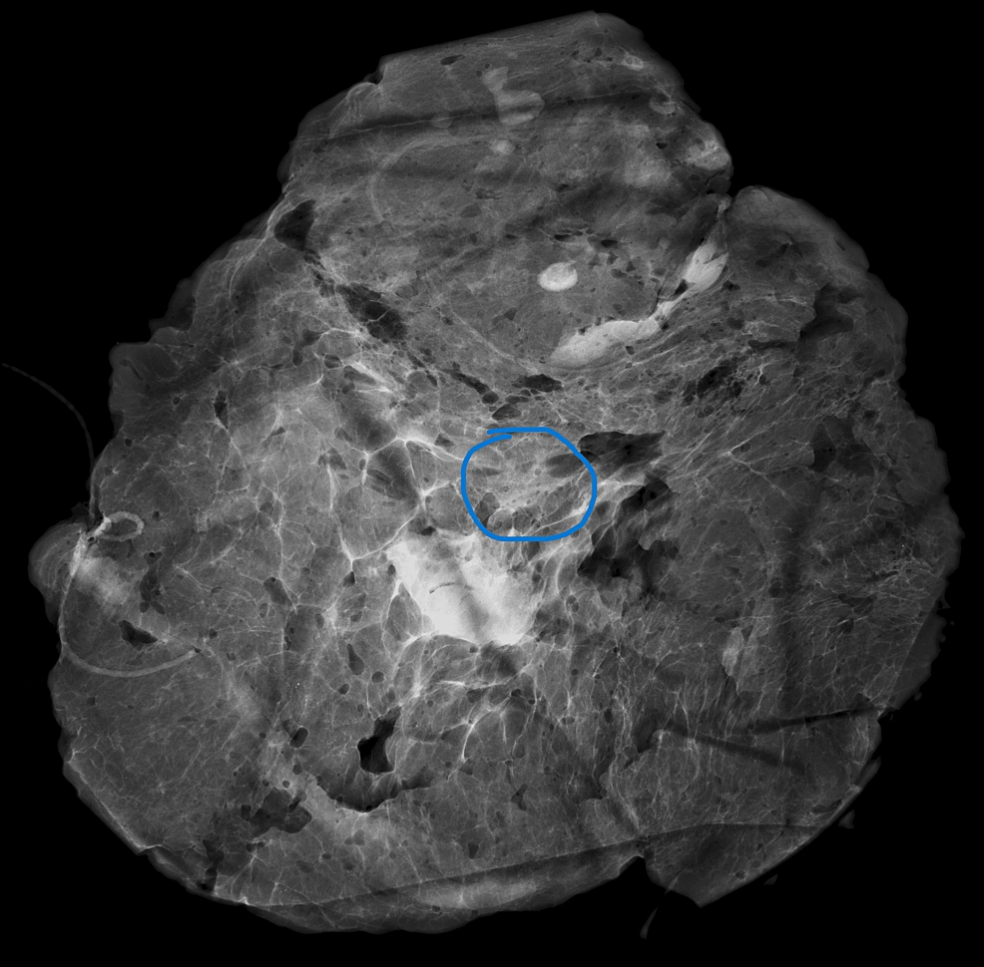
Case two mammogram showing an ill-defined area of the inner quadrants.

Case two: A 43-year-old Caucasian female with normal left breast palpation and a 4b BI-RADS due to a vague nodular, ill-defined area of the inner quadrants. Histological analysis showed fibrocystic changes (Figure 5).

**Figure 6:**
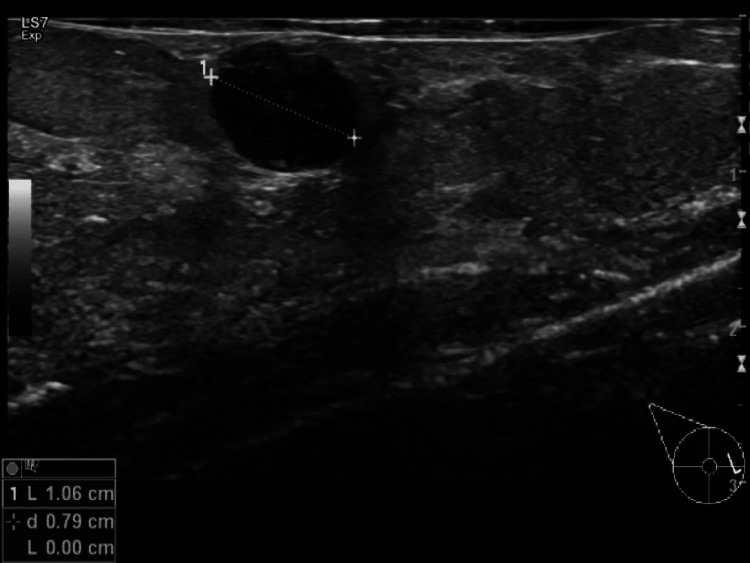
Case three ultrasound showing a nodular lesion in the inner quadrants.

Case three: A 55-year-old Caucasian female with a pathologic right breast palpation and 4b BI-RADS due to a nodular lesion in the inner quadrants (Figure 6).

Histology evidence showed there was a 10 mm steatonecrosis area.

Case four: A 57-year-old Caucasian female with normal right breast palpation and 4a BI-RADS due to a 0.8 mm nodular lesion in the inner quadrants. Histology, it was found to be a simple fibroadenoma.

Case five: A 75-year-old Caucasian female with a pathologic left breast palpation and 4b BI-RADS due to an external quadrant nodular lesion associated with microcalcifications. Histologically, there was a 10 mm calcified fibroadenoma and intraductal microcalcification.

Case six: A 76-year-old Caucasian female with a pathologic right breast palpation and 4a BI-RADS because of a nodular lesion in the central quadrants associated with macrocalcifications. Histologically, a 25 mm partially calcified cyst was found.

Case seven: A 79-year-old Caucasian female with a pathologic right breast palpation and 4a BI-RADS due to a nodular lesion in the external quadrants associated with macrocalcifications. Histologically, there was a 25 mm hamartoma.

Case eight: An 80-year-old Caucasian female with normal left breast palpation and 4a BI-RADS due to a nodular lesion in the external quadrants associated with macrocalcifications. Histology showed fibrocystic changes. No other biopsy was performed, and no silent breast cancer was detected.

## Discussion

A forensic autopsy is a postmortem examination performed to address medicolegal issues [11]. Historically, autopsies have served to answer questions inherent to medical care (diagnosis, quality assurance, and patient care), medical science and investigation (research, education, transplantation, and prostheses), society (public health, statistics, and forensics), and the family (counseling and understanding the life cycle) [12]. Aside from medicolegal or forensic autopsies, a new term has emerged, namely, research autopsies, which are performed primarily for collecting one or more normal or diseased tissues to support basic or translational research [13]. According to Iacobuzio-Donahue et al. [13], research autopsies are an underused approach to investigating the fundamental questions in cancer biology and hold tremendous potential in precision medicine.

In this study, the objective was to define the reservoir of breast cancer in serial, systematic, and research-oriented autopsies (systematic complete and thorough excision of breast and axillary content) of individuals who were not supposed to die and for whom assisting physicians could not find a cause of death (excluding in-hospital deaths even more biased by age ranges). The collected tissues were processed by imaging rather than by systematic histological examination. The systematic imaging (mammography and ecography) of breast glands is what distinguishes our approach. In other words, this study was designed to simulate an extended screening examination performed by breast disease dedicated professionals in the serial analysis of individuals. An obvious step would be to collect samples and verify the presence of pre/malignant tissue changes. We intended to suppress the overdiagnosis issue because were aware of it.

The latest published systematic review/meta-analysis on autopsy-detected breast cancer pointed out that incidental breast cancer and its precursors are widespread in women who have never been diagnosed with breast cancer, and that the vast pool of undiagnosed carcinoma in situ and atypical hyperplasia in these autopsy studies suggests that screening programs should be used with caution [14]. This study evidenced that the overall incidental cancer and precursor prevalence was as follows: invasive: 0.8%, in-situ: 8.9% (adjusted), and atypical hyperplasia: 9.8% (adjusted), for a total of 19.5%. In conclusion, autopsy samples, studied by histology, present a small reservoir (almost 1%) of invasive versus a large reservoir of in situ premalignant lesions (almost 18%). Hence, the incidental disease exists, but it is not detected by screening methods, even if they are extended to include ultrasound scanning of the breast tissue. As demonstrated by the null hypothesis, imaging techniques used for breast cancer screening do not overdiagnose the disease.

Breast population-based screening is intended to detect breast cancer at an early stage to enable lower mortality rates [15]. Three separate meta-analyses demonstrated a statistically significant (18-20%) reduction in mortality among women who were invited to the screening [16]. An overall estimate of various studies is that the mean reduction in mortality across all models is 15%, with the greatest reduction (39.6%) realized in the model initiating annual screening at age 40 [17]. Most societies making recommendations about breast cancer screening consider overdiagnosis as a substantial disadvantage. Overdiagnosis refers to the potential for overdetection of disease in asymptomatic women who are screened, which ultimately leads to overtreatment; in other words, diagnosing and treating breast cancer that would otherwise not threaten a woman’s health or longevity [18]. Various autopsy studies have attempted to define the natural reservoir of the disease to highlight the contribution of screening in the issue of overdiagnosis. Malignant and premalignant lesions in reduction mammoplasty specimens are expected to be between 1.5% and 14% in patients with no history of breast cancer [19]. This study did not support the above conclusions. Although the incidental disease exists, it is not detected by the screening methods (mammography) used in this study, even if they are extended and include ultrasound scanning of the breast tissue.

Limitations

The present research is subject to several limitations. First, the sampling number question is allocated. Because it was hypothesized that the prevalence of silent breast cancer is unknown and the actual disease incidence is low, finding a case of silent male breast cancer would be quite unusual. This limitation becomes a strength in the case of the female gender because contrary to what is believed, imaging sampling does not identify more malignancies than actually detected. Another limitation of this study is that medical data from the analyzed corpses could not be collected, leaving out potentially harmful or protective factors that would have been very interesting to investigate. The third and perhaps the most obvious limitation of this study is that specimens were not examined through systematic histology. This limitation stems from the study’s somewhat unique design that aimed to identify imaging-detected silent breast cancer, which breast screening has been shown to overdiagnose. Future directions should point to a combined autopsy study, which would include a large number of glands and compare imaging findings to the histology analyses. Such a study can, in the end, provide an unblemished answer to the following question: In what grade does breast cancer screening over-detect the disease?

## Conclusions

In light of the findings, it cannot be concluded that the imaging-detected silent breast cancer prevalence is higher than the actual incidence of the disease, contrary to the author’s initial hypothesis. Benign breast alterations were common, accounting for 43.6% of the corpses collected, while low-suspicion alterations were discovered in 1.84% of breast samples. The OE, which included inspection and palpation, missed 37.5% of the biopsied breast changes. This finding indicated that an OE presenting a significant number of false-negative results could not be used as a screening method. Because of the coronavirus disease 2019 pandemic-related suspension of sample collection, the study did not achieve the initial sample size needed to define the imaging-detected silent breast female cancer prevalence, and probably there would be identified individuals with breast silent cancer. Although because the null hypothesis was achieved, the result would not be statistically significant.
